# Zn(II) mediates vancomycin polymerization and potentiates its antibiotic activity against resistant bacteria

**DOI:** 10.1038/s41598-017-04868-2

**Published:** 2017-07-07

**Authors:** Ashraf Zarkan, Heather-Rose Macklyne, Dimitri Y. Chirgadze, Andrew D. Bond, Andrew R. Hesketh, Hee-Jeon Hong

**Affiliations:** 1 Department of Biochemistry, University of Cambridge, Cambridge, CB2 1QW UK; 2 Department of Chemistry, University of Cambridge, Cambridge, CB2 1EW UK; 3 Cambridge Systems Biology Centre, University of Cambridge, Cambridge, CB2 1QW UK; 4 Department of Biological and Medical Sciences, Oxford Brookes University, Oxford, OX3 0BP UK; 5 Department of Genetics, University of Cambridge, Cambridge, CB2 3EH UK

## Abstract

Vancomycin is known to bind to Zn(II) and can induce a zinc starvation response in bacteria. Here we identify a novel polymerization of vancomycin dimers by structural analysis of vancomycin-Zn(II) crystals and fibre X-ray diffraction. Bioassays indicate that this structure is associated with an increased antibiotic activity against bacterial strains possessing high level vancomycin resistance mediated by the reprogramming of peptidoglycan biosynthesis to use precursors terminating in D-Ala-D-Lac in place of D-Ala-D-Ala. Polymerization occurs via interaction of Zn(II) with the N-terminal methylleucine group of vancomycin, and we show that the activity of other glycopeptide antibiotics with this feature can also be similarly augmented by Zn(II). Construction and analysis of a model strain predominantly using D-Ala-D-Lac precursors for peptidoglycan biosynthesis during normal growth supports the hypothesis that Zn(II) mediated vancomycin polymerization enhances the binding affinity towards these precursors.

## Introduction

Antibiotics are a diminishing resource, with the emergence and spread of resistance in bacterial populations currently outstripping the pace of discovery and approval of novel compounds. There is consequently a renewed interest in finding leads for novel antibacterial activities, and for the development of methods capable of enhancing the effectiveness of existing treatments.

Vancomycin, a natural product glycopeptide antibiotic, is a last resort treatment for serious infections caused by Gram-positive bacterial pathogens, particularly those associated with MRSA (Methicillin resistant *Staphylococcus aureus*) or drug-resistant enterococci. The emergence of vancomycin resistance in these pathogenic strains is however threatening to reduce its usefulness as a front-line therapy^1, 2^. Vancomycin acts by binding to the D-Ala-D-Ala termini of bacterial cell wall peptidoglycan (PG) and of the PG precursor Lipid II, thereby compromising the synthesis and strength of the bacterial cell wall and leading to reduced growth and cell death^3^. A small number of semisynthetic second-generation lipoglycopeptide antibiotics have recently been developed to improve the treatment options available for Gram-positive infections, but possess the same mode of action as the parent template glycopeptide vancomycin^4, 5^.

Vancomycin has recently been characterized as a zinc chelator, binding Zn(II) via the N-terminal methylleucine amino acid group and inducing a zinc starvation response in treated bacteria^6, 7^. Although it is not common, some antibiotics are known to require metal ions for their activity^8^. Bacitracin requires binding of divalent metal ions for sequestration of its molecular target undecaprenyl pyrophosphate, with Zn(II) being most effective at stabilizing complex formation through neutralization of the pyrophosphate charge^9^. Quinolone antibiotics also use metal ions to help bind their target molecules, DNA topoisomerase enzymes^10, 11^. When not required to mediate target binding, interaction of metal ions with antibiotics can provide an additional mode of antibacterial action, as seen for example with the sequestration of iron by tetracyclines^12^. Here we report for the first time that zinc potentiates the antibiotic activity of vancomycin, and certain other glycopeptide antibiotics, specifically against bacterial strains exhibiting inducible glycopeptide resistance. Structural analysis indicates a novel polymerized arrangement of vancomycin molecules resulting from the interaction with Zn(II), and we use data from *in vitro* and *in vivo* studies to propose a new mode of action for this vancomycin polymer. We consider the significance of the interaction with Zn(II) for patient treatment.

## Results

### Zn(II) potentiates the activity of vancomycin against inducibly resistant bacteria

**Figure 1:**
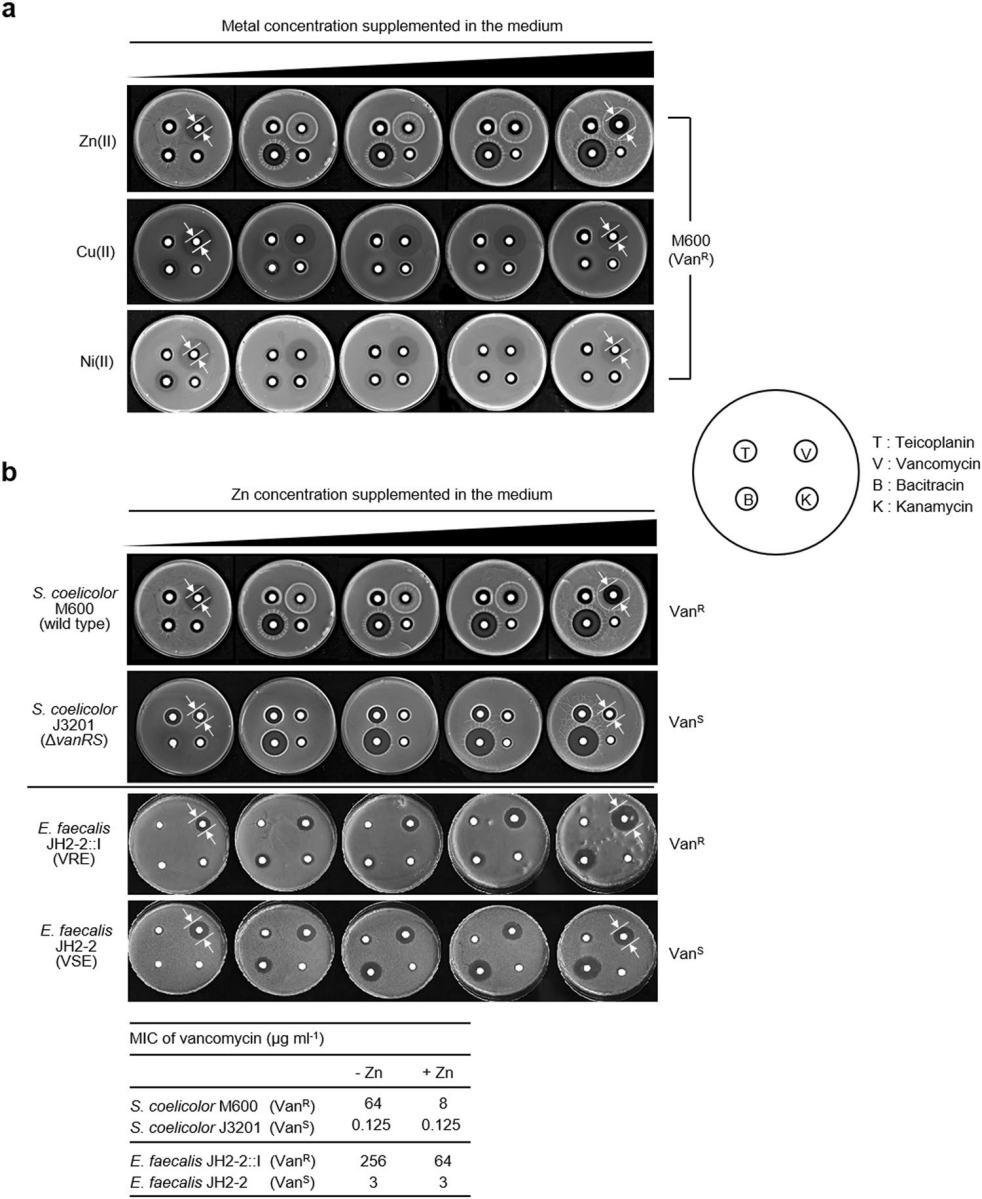
Zinc potentiates antibiotic activity of vancomycin against vancomycin-resistant bacteria. (**a**) The effect of Zn(II), Cu(II) and Ni(II) on the activity of teicoplanin (T), vancomycin (V), bacitracin (B) and kanamycin (K) against wild-type *S. coelicolor* M600. (**b**) The effect of Zn(II) on the activity of teicoplanin (T), vancomycin (V), bacitracin (B) and kanamycin (K) against vancomycin-sensitive (Van^S^) or vancomycin-resistant (Van^R^) strains of *S. coelicolor* and *E. faecalis*. The concentration of each antibiotic used is listed in Supplementary Table 1. The table shows the effect of Zn(II) on MIC values (μg ml^−1^) for vancomycin in resistant and sensitive strains.

To investigate the effect of the interaction of Zn(II) with vancomycin on antibiotic activity, we performed susceptibility tests using strains known to be inducibly resistant to vancomycin, and isogenic relatives lacking this resistance. *Streptomyces coelicolor* M600^13^ and *Enterococcus faecalis* JH2-2::I^14^ both possess dedicated resistance genes which direct the synthesis of modified PG precursors ending in D-Ala-D-Lac, to which glycopeptide antibiotics exhibit very low binding affinities. Expression of resistance is induced by vancomycin via a two-component regulatory system composed of a sensor kinase (VanS) and a cognate response regulator (VanR) that acts as a transcriptional activator when phosphorylated. Strikingly, co-administration of vancomycin with increasing concentrations of zinc sulphate (0-200 μM) increased the zone of growth inhibition against vancomycin-resistant *S. coelicolor* M600 in a paper disc diffusion assay (Fig. 1a). This effect is specific for Zn(II). Both Ni(II) and Cu(II), the latter known to more strongly interact with vancomycin than Zn(II)^7, 15, 16^, had no effect on the activity of vancomycin. As expected, the presence of Zn(II) also enhanced the antibiotic activity of bacitracin^9^, but did not influence kanamycin (Fig. 1a). 200 μM Zn(II) also failed to increase the antibacterial activity of ramoplanin, moenomycin, novobiocin, apramycin and tunicamycin against *S. coelicolor* M600 (Supplementary Fig. 1), indicating that the concentration of Zn(II) used in the experiments are in the non-toxic range. Teicoplanin, a naturally occurring lipoglycopeptide antibiotic, also showed no increase in activity in the presence of Zn(II) (Fig. 1a). Importantly, the potentiation of vancomycin activity by Zn(II) was also observed when using *E. faecalis* JH2-2::I as the test strain indicating that this is a more general phenomenon (Fig. 1b). Minimal inhibitory concentration (MIC) measurements attribute a 4- to 8-fold increase in susceptibility towards vancomycin to Zn(II) in *E. faecalis* JH2-2::I and *S. coelicolor* M600, respectively (Fig. 1b). Notably, the use of the vancomycin-sensitive strains *S. coelicolor* J3201^17^ and *E. faecalis* JH2-2^14^ abolished the influence of Zn(II) on vancomycin activity, but had no abrogating effect on the enhancement of bacitracin activity (Fig. 1b). The VanRS two-component regulatory system required for inducing vancomycin resistance is deleted in *S. coelicolor* J3201, while *E. faecalis* JH2-2 lacks the transposon carrying all the vancomycin resistance genes that is present in JH2-2::I. This implies that the potentiation of vancomycin by Zn(II) is directly related to the functioning of the vancomycin resistance system.

### Only glycopeptide antibiotics which are capable of activating inducible resistance are potentiated by Zn(II)

**Figure 2:**
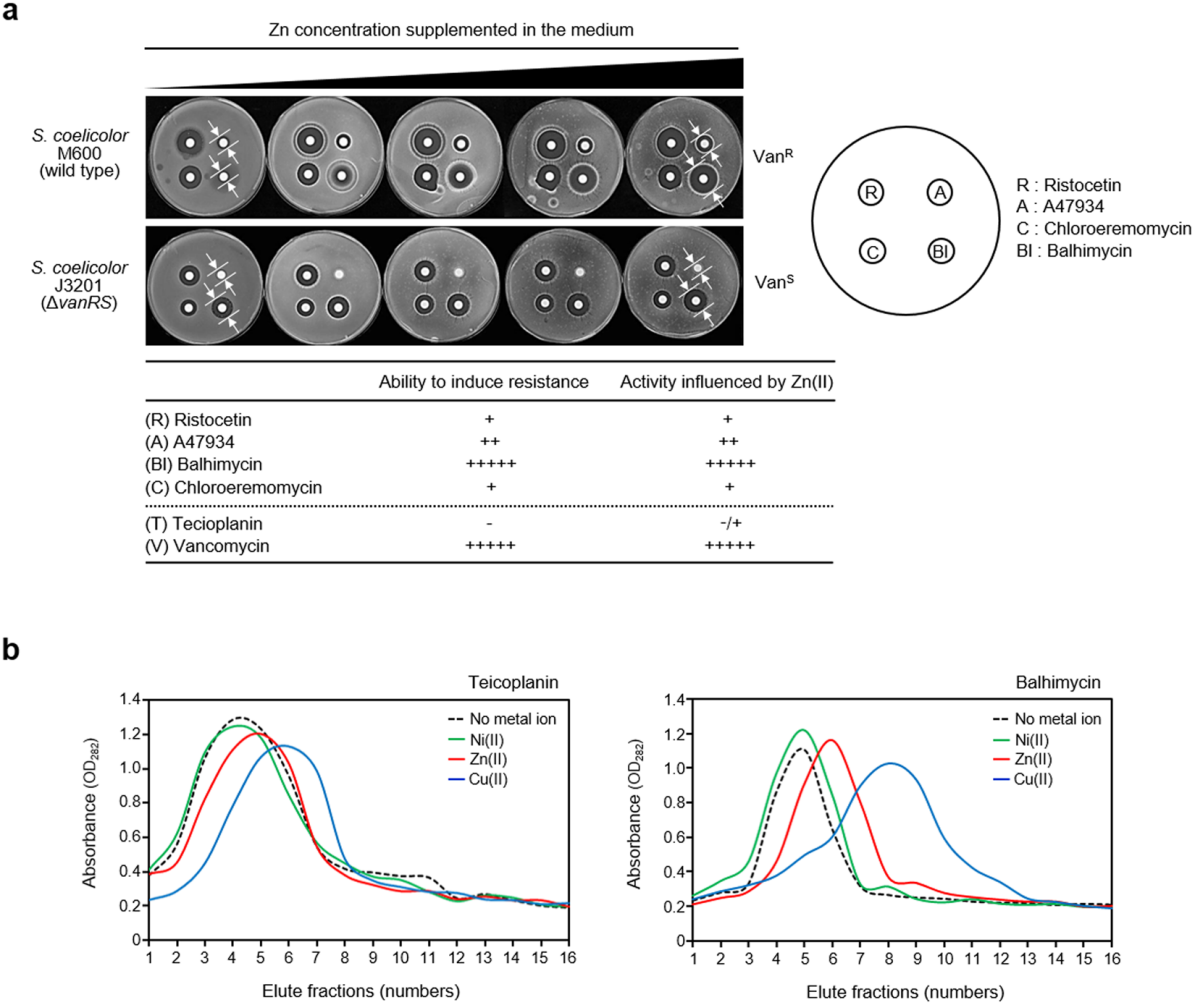
Zn(II) potentiates the activity of glycopeptide antibiotics that are both strong inducers for resistance and which interact with Zn(II). (**a**) The effect of Zn(II) on the activity of ristocetin (R), A47934 (A), chloroeremomycin (C) and balhimycin (Bl) against vancomycin-resistant and -sensitive *S. coelicolor* strains. The table summarizes the bioassay results shown above, presenting the influence of Zn(II) on antibiotic activity against an index summarizing the ability of each antibiotic to induce the VanS sensor in *S. coelicolor*, obtained from previous studies^13, 17–21^. (**b**) Metal affinity chromatography to measure the interaction of balhimycin and teicoplanin with Zn(II), Cu(II) or Ni(II). Elution of teicoplanin or balhimycin from the columns was quantified by the analysis of 0.5 ml fractions at OD_282_ and a bioassay against a vancomycin sensitive *S. coelicolor* ∆*vanRS* mutant strain (Supplementary Fig. S4).

To define the relationship between the effect of Zn(II) on glycopeptide antibiotic activity and induction of glycopeptide resistance, a broader panel of natural glycopeptides were assayed for activity in different streptomycete indicator strains (Fig. 2a and Supplementary Fig. 2). Glycopeptide-strain combinations where the strength of induction via the VanS sensor kinase had previously been characterized^13, 17–21^ were selected, and assayed for any potentiation by Zn(II). In strains possessing functional inducible glycopeptide resistance systems, Zn(II)-dependent enhancement of glycopeptide antibiotic activity was closely correlated with the ability of the glycopeptide to act as an inducer of glycopeptide resistance. Thus in *S. coelicolor* M600, balhimycin and vancomycin are both the strongest inducers and exhibit the most marked potentiation of activity by Zn(II). In contrast, poor inducers of VanS in this strain background i.e. ristocetin, A47934, chloroeremomycin, and teicoplanin show only small or negligible changes in activity on addition of Zn(II) (Fig. 2a). This correlation is confirmed by observations in *Streptomyces toyocaensis*, where, in contrast to *S. coelicolor* M600, differences in the amino acid sequence of the VanS sensor kinase (VansSst) dictate that vancomycin is a poor inducer of resistance and A47934 the strongest inducer (Supplementary Fig. 2)^19–21^. The influence of Zn(II) on the activity of these antibiotics is also reversed between the two strains, with vancomycin activity against *S. toyocaensis* no longer affected by Zn(II) but the activity of A47934 being most markedly increased. Also consistent with these observations, control strains lacking any inducible glycopeptide resistance, *S. coelicolor* J3201 and *Streptomyces avermitilis*, exhibited no interaction between Zn(II) and glycopeptide activity (Fig. 2a and Supplementary Fig. 2).

Bioassay results indicate that in the *S. coelicolor* M600 background the activities of vancomycin and balhimycin are each capable of being enhanced by Zn(II) (Fig. 2a). Vancomycin is known to bind Zn(II)^7^. To determine whether balhimycin can participate in a similar physical interaction, affinity column chromatography, fluorometry and Calf Intestinal Alkaline Phosphatase (CIAP) inhibition assays were performed as previously described for vancomycin^7^ (Fig. 2b and Supplementary Figs 3–5). All three methods indicated an interaction with Zn(II) that is notably stronger than that observed for teicoplanin whose antibiotic activity is influenced only marginally by Zn(II). Zn(II)-dependent enhancement of glycopeptide activity therefore appears to require both the ability of the antibiotic to induce glycopeptide resistance via VanS, and physical interaction with Zn(II).

### The potentiation of vancomycin activity by Zn(II) requires remodelling of PG termini to D-Ala-D-Lac

**Figure 3:**
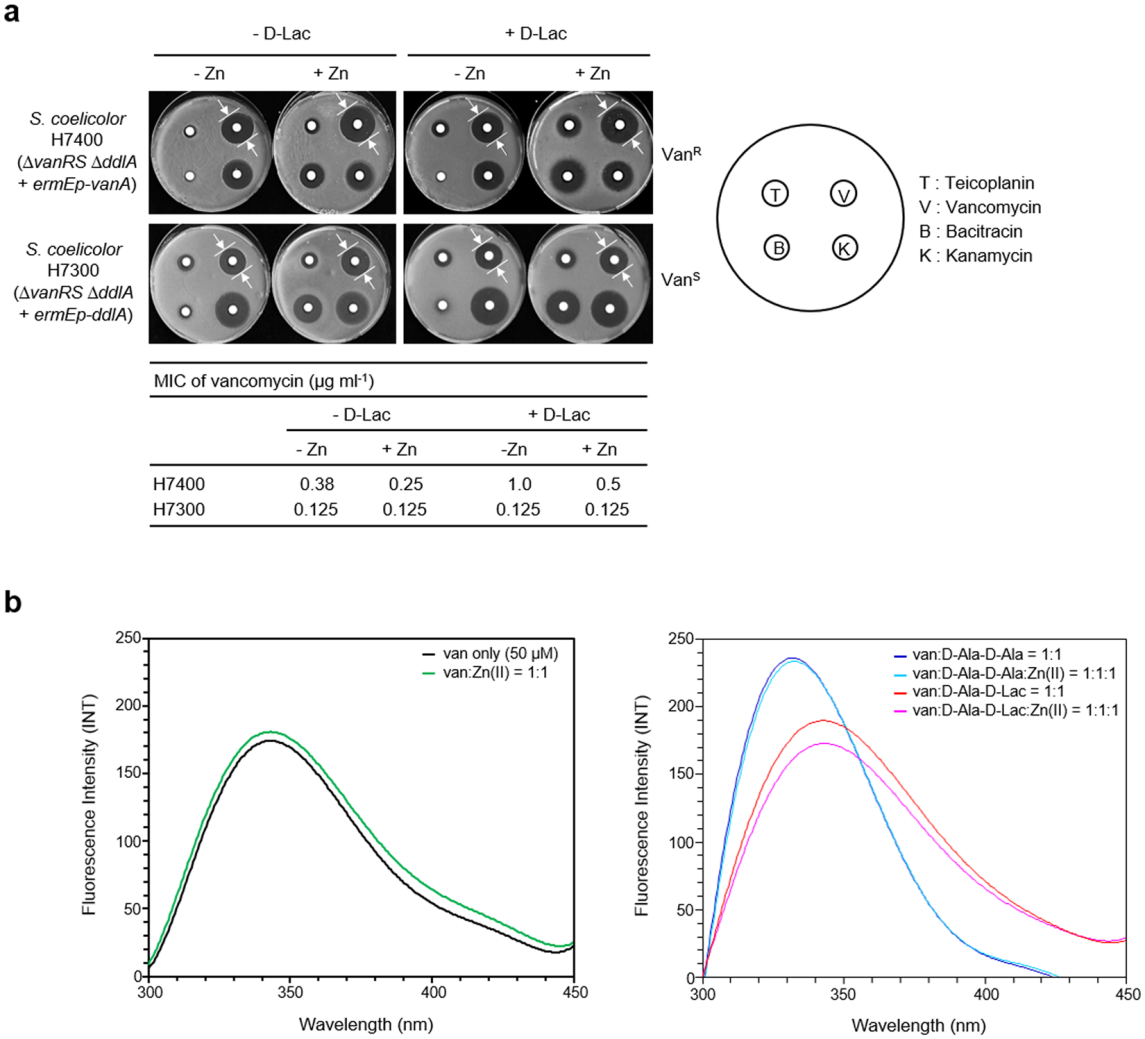
Interaction of Zn(II) with vancomycin increases its activity against glycopeptide-resistant peptidoglycan precursors terminating in D-Ala-D-Lac, and changes its binding. (**a**) The effect of Zn(II) on the activity of teicoplanin (T), vancomycin (V), bacitracin (B) and kanamycin (K) against *S. coelicolor* strains engineered to constitutively produce peptidoglycan precursors terminating in D-Ala-D-Lac (H7400) or D-Ala-D-Ala (H7300). Supplementation with D-lactate (D-Lac; 10 mM) promotes the major D-Ala-D-Lac ligase activity of VanA at the expense of the minor D-Ala-D-Ala activity. (**b**) Fluorometric analysis indicating a change in the interaction between vancomycin and D-Ala-D-Lac, but not D-Ala-D-Ala, in the presence of Zn(II). The left panel shows the effect of Zn(II) on vancomycin fluorescence for comparison.

Three different hypotheses could explain the effect of Zn(II) on vancomycin (and other glycopeptide) antibiotic activity in inducibly resistant bacterial strains, and these were tested experimentally. An inhibitory effect on the Zn(II)-dependent D-Ala-D-Ala dipeptidase activity of the VanX vancomycin resistance enzyme was ruled out by analysis of the *S. coelicolor* ∆*vanX* mutant J3225^13^ which was found to respond to Zn(II)-vancomycin in the same way as the M600 parent strain (Supplementary Fig. 6). The hypothesis that interaction of vancomycin with Zn(II) increased sensitivity by inhibiting activation of the VanS sensor kinase by the glycopeptide was also eliminated through the analysis of mutant strains (Supplementary Fig. 7). *vanS* mutants of *S. coelicolor* (J3200)^17^ and *E. faecalis* (JH2-2::C1)^14^ which exhibit constitutively active glycopeptide resistance, with no requirement for the presence of antibiotic inducer, again show a normal enhancement of vancomycin activity in response to Zn(II) (Supplementary Fig. 7). The remaining possibility is that interaction of vancomycin with Zn(II) increases the inhibition of PG biosynthesis that takes place using the remodelled vancomycin-resistant PG precursors possessing D-Ala-D-Lac termini. To investigate this hypothesis we exploited the activities of the homologous enzymes VanA and DdlA in *S. coelicolor*. VanA is a non-essential bifunctional D-Ala-D-Ala and D-Ala-D-Lac ligase enzyme which preferentially synthesizes D-Ala-D-Lac in the presence of D-Lactate (D-Lac), and supplies the substrate required for the remodelling of PG biosynthesis in response to vancomycin^13, 22^. In contrast, DdlA encodes the essential D-Ala-D-Ala ligase activity responsible for cell wall formation during normal growth using PG precursors with D-Ala-D-Ala termini^22, 23^. To generate a strain (H7400) constitutively using PG terminating in D-Ala-D-Lac during normal growth, we introduced the *vanA* gene under the control of the strong and constitutive *ermE* promoter (*ermEp*) into the *S. coelicolor* ∆*vanRS* mutant J3201, then successfully deleted the normally essential *ddlA* gene. An analogous strain (H7300) expressing *ddlA* from the *ermEp* was also generated as a control. Use of the ∆*vanRS* mutant background ensures that there is no contribution from the native vancomycin-inducible *vanA* gene. Vancomycin antibiotic activity was potentiated by Zn(II) only in strain H7400 where PG precursors terminating in D-Ala-D-Lac are used for cell wall biosynthesis, not in H7300 which uses PG precursors terminating in D-Ala-D-Ala (Fig. 3a). This effect was further enhanced when the growth medium was supplemented with D-Lactate to promote the D-Ala-D-Lac ligase activity of VanA (Fig. 3a). This implies that the interaction with Zn(II) can specifically increase the binding affinity of vancomycin for PG molecules terminating in D-Ala-D-Lac. This interpretation is supported by *in vitro* fluorometric analysis which shows a marked quenching of vancomycin fluorescence on addition of Zn(II) to a solution containing vancomycin and D-Ala-D-Lac, but not vancomycin in the presence of D-Ala-D-Ala (Fig. 3b). In contrast, addition of Zn(II) to vancomycin enhances fluorescence in the control indicating different binding (Fig. 3b).

### Zn(II) mediates polymerisation of vancomycin

**Figure 4:**
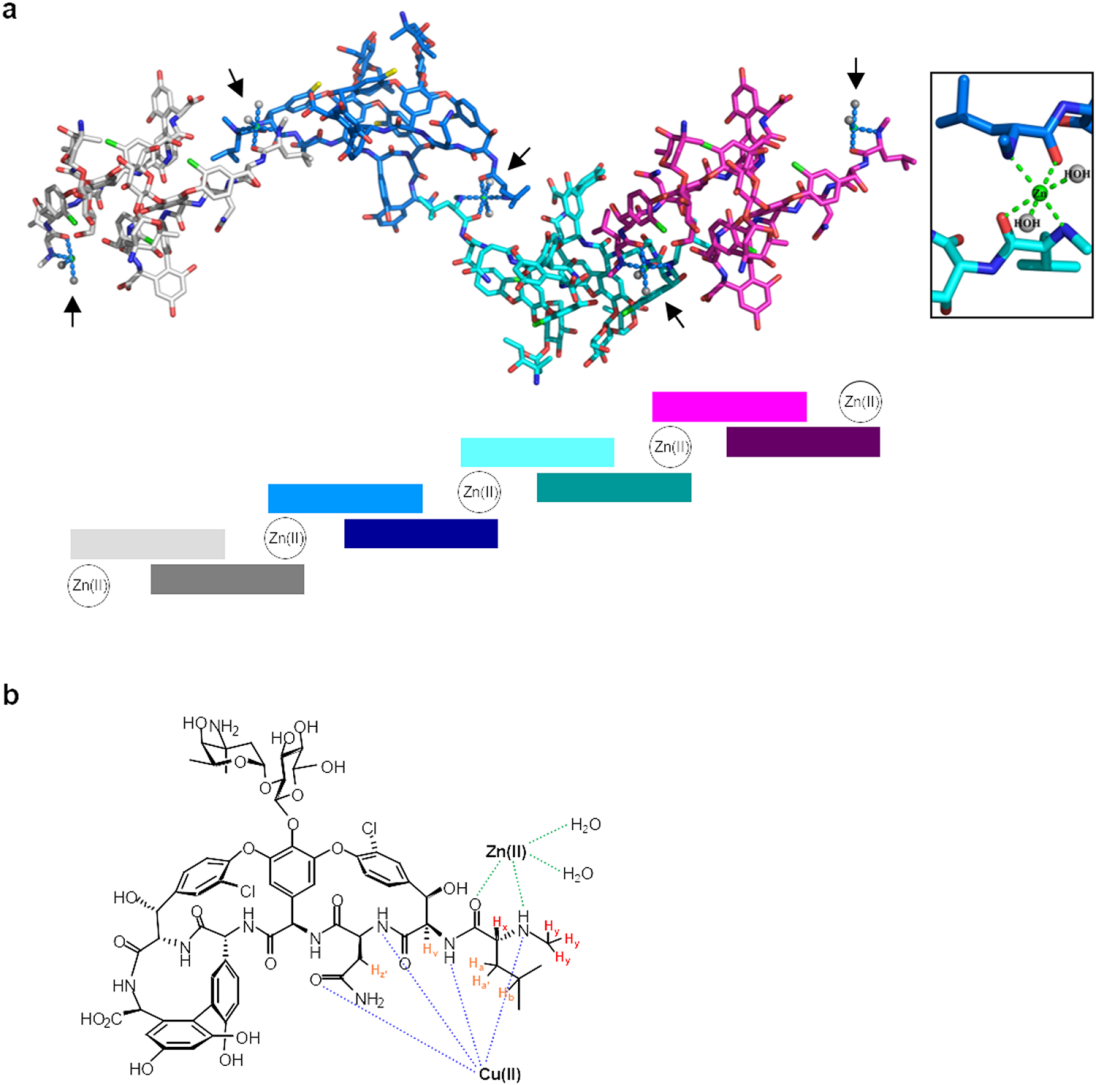
Zn(II) interacts with the N-terminal methylleucine of vancomycin to produce a novel polymer of dimers. (**a**) Crystal structure of vancomycin-Zn(II) determined by X-ray crystallography. Zn(II) ions (indicated by arrows) hold dimers of vancomycin in a polymeric structure, as also illustrated in the schematic representation showing the arrangement of vancomycin molecules (coloured rectangles, stacked in dimers) and Zn(II) ions in the polymer. The inset shows Zn(II) joining adjacent vancomycin dimers via ionic interaction with the carbonyl (in red) and secondary amine (in blue) groups of the N-terminal methylleucine group. (**b**) Chemical structure of vancomycin indicating the site of interaction with Zn(II) relative to Cu(II).

The interaction of vancomycin with Zn(II) is associated with a novel D-Ala-D-Lac binding activity. To investigate the structural basis for this activity we have crystallized the vancomycin-Zn(II) complex. Our first attempt generated a crystal structure for a vancomycin-Zn(II)-citrate complex containing two independent vancomycin molecules in an asymmetric unit with space group C222, plus a separate complex of Zn(II) coordinated by two citrate molecules and a polyethylene glycol molecule (Supplementary Fig. 8a and Supplementary Table 2). This dimeric arrangement of vancomycin is identical to that previously reported by Schäfer and colleagues (space group P 4_3_2_1_2) (Supplementary Fig. 8c)^24^. Subsequent crystallization in the absence of citrate successfully generated a clean vancomycin-Zn(II) crystal structure (Fig. 4a and Supplementary Fig. 8b). The structure revealed two independent vancomycin molecules in space group H3, and a Zn(II) ion coordinated by two water molecules and two atoms derived from the N-terminal methylleucine group of vancomycin, including one amino nitrogen and one carboxyl oxygen (Fig. 4a, Supplementary Fig. 8b and Supplementary Table 2). This binding site for Zn(II) to the vancomycin molecule is entirely consistent with that predicted from our previous NMR solution structural studies, and is distinct from the binding site for Cu(II) (Fig. 4b)^7, 15^. The presence of Zn(II) allows vancomycin to adopt a novel conformation not previously observed in reported vancomycin crystal structures (Supplementary Fig. 8b), and is distinct from the conformation of the vancomycin-Cu(II) complex in which two independent vancomycin molecules are arranged in space group C2 (Supplementary Fig. 8d)^16^. This novel conformation allows the assembly of repeated vancomycin dimers held together by coordination with Zn(II) to generate a vancomycin polymer (Fig. 4a). Fibre X-ray diffraction, a technique to study the molecular structure of long assemblies of identical subunits^25^, confirmed the polymerization of vancomycin by Zn(II) (Supplementary Fig. 9). Analysis of a sample containing both vancomycin and Zn(II) generated a diffraction pattern consisting of three concentric reflection rings at 25 Å, 11 Å and 8 Å of Braggs’ interplanar distances, consistent with the presence of repetitive polymer fibres with a low degree of alignment^26, 27^. These were completely absent in similar analysis of a control sample of vancomycin lacking Zn(II) (Supplementary Fig. 9).

## Discussion

High-level glycopeptide resistance in Gram-positive bacteria is due to the replacement of the D-Ala-D-Ala terminus of PG precursors with D-Ala-D-Lac, to which the antibiotics exhibit lower binding affinities^4^. Replacement with D-Ala-D-Ser can also occur but generates markedly lower resistance due to production of a smaller defect in antibiotic binding. The identification of a lead glycopeptide structure with increased activity against cell wall biosynthesis proceeding via PG precursors with D-Ala-D-Lac termini is therefore of significant interest. The novel polymerization of vancomycin mediated by interaction with Zn(II) is associated with increased antibiotic activity against model D-Ala-D-Lac-based glycopeptide resistant strains, including an *E. faecalis* VRE strain. Interestingly, a synthetic polymer of vancomycin monomers with enhanced potency against VRE has previously been reported, and surface plasmon resonance studies also indicated an increased binding affinity between the polymer and a VRE cell wall mimic containing D-Ala-D-Lac^28^. In this study, Zn(II) causes polymerization of vancomycin dimers, with the metal ion holding repeated dimeric units in place via interaction with nitrogen and oxygen atoms in the N-terminal methylleucine moiety (Fig. 4).

Other glycopeptide or lipoglycopeptide antibiotics which possess the methylleucine interaction site would also be expected to bind Zn(II) and may also be susceptible to similar polymerization and enhancement of antibiotic activity. Consistent with this, Zn(II) was able to increase the activity of two antibiotics with the methylleucine group, balhimycin and A47934, but had no effect on either teicoplanin or ristocetin which lack this feature (Supplementary Table 3). *In vitro* assays also demonstrated markedly stronger binding of Zn(II) to balhimycin compared to teicoplanin. Chloroeremomycin does possess a potential binding site but Zn(II) did not produce an effect in a bioassay against *S. coelicolor* M600 since the antibiotic is not a good inducer of the D-Ala-D-Lac resistance system. Interestingly, of the three new semi-synthetic lipoglycopeptide antibiotics being brought into clinical use, both oritavancin and telavancin retain the methylleucine present in vancomycin and therefore potentially also retain the ability to interact with Zn(II). Dalbavancin has a structure related to teicoplanin and would not be predicted to interact.

Due to the well-known effect of Zn(II) on bacitracin antibiotic activity, topical treatments for application to wound infections come pre-formulated as bacitracin-zinc. The results of this study suggest that a similar approach may also be beneficial for vancomycin to augment activity against vancomycin resistant pathogens. This could be evaluated for clinical significance using a suitable infection model. Some of the known side effects of vancomycin in long term treatments also coincidently overlap with aspects of zinc deficiency syndrome, such as nephrotoxicity or muscle spasm^29, 30^. Careful co-administration of vancomycin with a Zn(II) additive could therefore be helpful for both antibiotic activity and the alleviation of toxic side effects. Further studies to determine the structure of the tertiary vancomycin-Zn(II)-D-Ala-D-Lac complex will be necessary to fully understand the nature of the binding to D-Ala-D-Lac PG precursors, and to be able to further exploit the enhanced affinity of polymerized vancomycin and other glycopeptide antibiotics toward D-Ala-D-Lac.

## Methods

### Bacterial strains, plasmids, oligos, antibiotics and culture conditions

The bacterial stains, plasmids and oligos used in this study are described and listed in Supplementary Table 4. A47934 was prepared from the producer strain *S. toyocaensis* as described previously^31^. Balhimycin and chloroeremomycin were kind gifts from Andy Truman. All other antibiotics used for this study were purchased from Sigma-Aldrich. Except where detailed below, media and culture conditions were as previously described^32^.

### Antibiotic susceptibility tests

All susceptibility tests, including the antibiotic disc diffusion assays and MIC determinations, were performed on MMCGT agar medium^33^ supplemented with the required concentration of metal sulphate. Results were scored after 2–4 days of incubation at 30 °C. For the antibiotic disc diffusion assay, for *S. coelicolor*, 0, 50, 100, 150 and 200 μM of metal sulphate was added to the media, and for *S. avermitilis* 0, 100 and 200 μM of metal sulphate was used. For *S. toyocaensis*, the assay was performed using 0, 50 and 100 μM of metal sulphate. *E. faecalis* strains were exposed to 0, 100, 200, 300 and 400 μM metal sulphate. Concentrations beyond these maximum levels can cause toxicity and were avoided. For the bioassay of all *Streptomyces* strains, approximately 10^7^ spores were used to seed the test plates. For the *E. faecalis* strains, approximately 10^7^ cfu from an overnight LB liquid culture were spread on the MMCGT medium. The concentrations of antibiotics used in the bioassays are listed in Supplementary Table 1. For the determination of the vancomycin MICs, Etest strips purchased from Biomerieux were used. All bioassays were performed at least three times and representative results are presented. The degree of relative variation for the inhibitory halo size in plate diffusion tests was less than 10%.

### Construction of *S. coelicolor* H7300 and H7400

*S. coelicolor* H7300 (∆*vanRS* ∆*ddlA* + *ermEp*-*ddlA*) and H7400 (∆v*anRS* Δ*ddlA* + *ermEp*-*vanA*), were constructed from *S. coelicolor* ∆v*anRS* J3201^17^ as follows. Initial transformation with pGN8 (pIJ10257 *ermEp*-*ddlA*)^22^ or pGN17 (pIJ10257 *ermEp*-*vanA*)^22^ generated strains H7100 and H7200, respectively. Deletion of the *ddlA* gene in H7100 and H7200 was then accomplished by PCR-directed mutagenesis as described previously^22^, creating H7300 and H7400, respectively.

### Affinity column chromatography

Metal affinity column chromatography experiments were performed as described previously^7^. HiTrap Chelating HP (GE healthcare) columns were initially washed with 5 ml of MilliQ water with a flow rate of 1 ml min^−1^ then loaded with 500 μl of 100 mM copper, nickel or zinc sulphate solutions. A column loaded with buffer solution was used as an uncharged negative control. Columns were washed again with 5 ml of MilliQ water then equilibrated with 10 ml of binding buffer (20 mM NaH_2_PO_4_/Na_2_HPO_4_ (pH 7.4), 200 mM NaCl, 10% Glycerol, 0.1% Triton-X-100). 150 μl of 10 mM balhimycin or teicoplanin was applied to each column and elution performed using 5 ml of binding buffer followed by 5 ml of binding buffer supplemented with 50 mM imidazole. Sixteen 0.5 ml fractions were collected (after discarding the dead volume of the column) and the relative abundance of antibiotic in each fraction was measured at OD_282_ after 5x dilution in water. Aliquots of the same diluted fractions were also analysed in a bioassay. For this, 20 μl was applied to freshly prepared MMCGT bioassay plates seeded with ~10^7^ spores of a vancomycin-sensitive *S. coelicolor* ∆*vanRS* strain (J3201)^17^. The results were scored after incubation at 30 °C for 2–4 days.

### Fluorometry

Fluorometric analysis was performed in 100 mM Tris-Cl (pH7.3) according to the method described previously^7^ using a PerkinElmer LS55 fluorescence spectrometer (Waltham, MA, USA) set to a 280 nm excitation wavelength; 5 nm slit width; 300–450 nm emission scan range; and 50 nm min^−1^ scan speed. Vancomycin solutions were supplemented with 50 µM of Ac-Lys(Ac)-D-Ala-D-Ala-OH or Ac-Lys(Ac)-D-Ala-D-Lac-OH (Bachem) where indicated. Zn(II) was added to the required concentration from a 50 mM stock solution of zinc sulphate heptahydrate in deionized water.

### Crystallization, data collection and refinement

Vancomycin hydrochloride (Sigma, V2002) and zinc sulphate heptahydrate (Sigma, Z4750) were purchased from Sigma and used without further purification. Vancomycin was used at a concentration of 50 mg ml^−1^ in 10 mM HEPES buffer at pH 7.0^22^, and Zn(II) added to a 10-fold molar excess, adjusting the pH to 7.0 as required. Any insoluble material was removed by centrifugation, and the supernatant was used to set up MRC 2-drop crystallisation trial plates containing conditions from the JCSG + (Molecular Dimensions) or PEGs I (Qiagen) crystallisation suites. Plates were loaded with two drops per reservoir, each containing 0.2 μl sample + 0.2 μl of crystallisation suite mix, using a Mosquito® crystallisation robot (TTP Labtech). The plates were then incubated at 19 °C in a Rock Imager Hotel/Imager (Formulatrix) and imaged (visual and UV) over a period of 34 days. Any crystals formed were harvested using 0.2–0.3 ml cryoloops (Hampton) and cryoprotectant solution containing 26% ethylene glycol and reservoir solution. Harvested crystals were plunged into liquid nitrogen and either stored or used for diffraction experiments straight away. Well A3 of the JCSG + plate, the reservoir of which contains 0.2 M di-ammonium hydrogen citrate salt [(NH_4_)_2_H Cit (C_6_H_5_O_7_)] + 20% (w/v) PEG 3350, produced the crystal found to be a tertiary vancomycin-Zn(II)-citrate complex. The desired vancomycin-Zn(II) crystal was obtained from well F8 of the PEGs I plate, from a reservoir comprising 0.2 M magnesium formate salt + 20% (w/v) PEG 3350. Diffraction data were collected at the Diamond Synchrotron Facility (Oxford, UK). A summary of the conditions for the data collection and the structure refinement parameters are given in Supplementary Table 2. The crystal structures were solved either by direct methods or using the single wavelength anomalous dispersion (SAD) method, with the chlorine atoms as the anomalous scatters (Cl-SAD). Model coordinates have been deposited in the Protein Data Bank (PDB) under accession number 5M2H for the vancomycin-Zn(II)-citrate complex and under accession number 5M2K for the vancomycin-Zn(II) complex.

### Fibre X-ray diffraction

X-ray diffraction studies were performed at the Crystallographic X-ray facility, Department of Biochemistry, University of Cambridge using an X8 Proteum (Bruker AXS). Fibre samples were placed in the X-ray beam and the diffraction data were collected with a sample-to-detector distance of 120 mm and an exposure time of 120 sec. Fibre samples for analysis were generated from solutions of vancomycin (5 mg ml^−1^ solution of vancomycin hydrochloride in 10 mM HEPES buffer at pH 7.0) or vancomycin-Zn(II) (vancomycin solution plus a 10-fold molar excess of zinc sulphate heptahydrate (Sigma, Z4750) in 10 mM HEPES buffer at pH 7.0) as described in the Supplementary Methods.

## Electronic supplementary material

Supplementary information
