# Supplementary material for: Zn(II) mediates vancomycin polymerization and potentiates its antibiotic activity against resistant bacteria
Source: Sci Rep. 2017 Jul 7;7:4893. doi: 10.1038/s41598-017-04868-2 (PMC5501778; doi:10.1038/s41598-017-04868-2)
Supplement: Supplementary file 1 — Supplementary information [file 41598_2017_4868_MOESM1_ESM.pdf]

SUPPLEMENTARY INFORMATION

Zn(II) mediates vancomycin polymerization and potentiates its antibiotic activity against resistant bacteria

Ashraf Zarkan[1,†], Heather-Rose Macklyne[1], Dimitri Y. Chirgadze[1], Andrew D. Bond[2], Andrew R. Hesketh[1,3], and Hee-Jeon Hong[1,4*]

[1]Department of Biochemistry, University of Cambridge, Cambridge, CB2 1QW, UK

[2]Department of Chemistry, University of Cambridge, Cambridge, CB2 1EW, UK

[3]Cambridge Systems Biology Centre, University of Cambridge, Cambridge, CB2 1QW, UK

[4]Department of Biological and Medical Sciences, Oxford Brookes University, Oxford, OX3 0BP, UK

[†]Present address: Department of Genetics, University of Cambridge, Cambridge, CB2 3EH, UK

*Correspondence: hee-jeon.hong@brookes.ac.uk

Supplementary Methods

Calf Intestinal Alkaline Phosphatase (CIAP) activity assay

To measure any binding between Zn(II) and selected glycopeptide antibiotics (bacitracin, kanamycin, balhimycin and teicoplanin), CIAP activity assays were performed as described previously[1]. CIAP is a metalloprotein which requires zinc as a cofactor for its activity[2-4]. CIAP dephosphorylates colourless p-nitrophenylphosphate (pNPP) to release the yellow coloured reaction product p-nitrophenol (pNP). The rate of colour evolution is proportional to the enzyme activity and can be quantified at 415 nm. To measure the effect of bacitracin, kanamycin, balhimycin or teicoplanin on CIAP enzyme activity aliquots (50 µl) of a concentration series of each antibiotic (0, 1, 2, 5, 10, 15, 20, 50, 100 mM) were added to aliquots (50 µl) of CIAP enzyme (0.1 units in the supplied reaction buffer; New England Bioloabs) in a 96 well plate then mixed with pNPP substrate (Sigma; 100 µl of a 0.5 mg ml^{-1} solution in 50 mM Tris-Cl (pH8)). pNP formation was quantified at 415 nm at regular intervals over 90 min using a Bio-Rad 680 Microplate Reader.

Fluorometry

Fluorometric analysis of the interaction of teicoplanin or balhimycin with metal ions was performed according to the method described previously[1] using a PerkinElmer LS55 fluorescence spectrometer (Waltham, MA, USA) set to a 280 nm excitation wavelength; 5 nm slit width; 300-450 nm emission scan range; and 50 nm min^{-1} scan speed. Zinc, copper, or nickel sulphate was added as required from 50 mM stock solutions in deionized water. Typically, 50 µM of each antibiotic in 100 mM Tris-Cl buffer (pH 7.3) was used.

Preparation of fibres for X-ray diffraction analysis

Fibres were prepared from solutions according to Morris and Serpell (2012)[5]. For each fibre, a glass capillary tube was dipped into melted bees wax, drawing in a small length (1-2 mm)

of wax at both ends of each tube. The tube was allowed to cool to solidify the wax before cutting in half to generate two individual capillaries each plugged with wax at one end. These were placed horizontally, secured to a petri dish using plasticine, with their wax-sealed ends facing each other at a distance of about 0.5 to 1 mm apart. A droplet of the solution to be analysed was carefully placed between the waxed ends of the aligned capillary tubes and allowed to evaporate. Loading and evaporation was repeated until visible solidified material was formed.

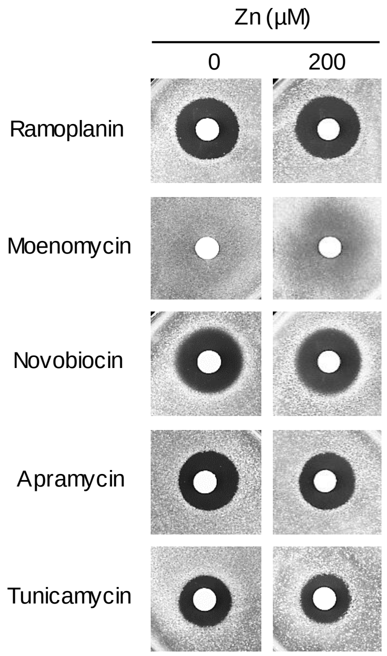

Supplementary Figure 1: The effect of Zn(II) on the activity of a range of non-glycopeptide antibiotics against *S. coelicolor* wild type M600. Approximately 10^7 spores of *S. coelicolor* M600 were spread onto MMCGT agar medium supplemented with 0 or 200 µM of zinc sulphate, then 10 µg of each antibiotic was applied to a paper disc. The result was scored after 2-4 days of incubation at 30ºC. The result indicates that Zn(II) does not positively influence the activity of any of the antibiotics tested.

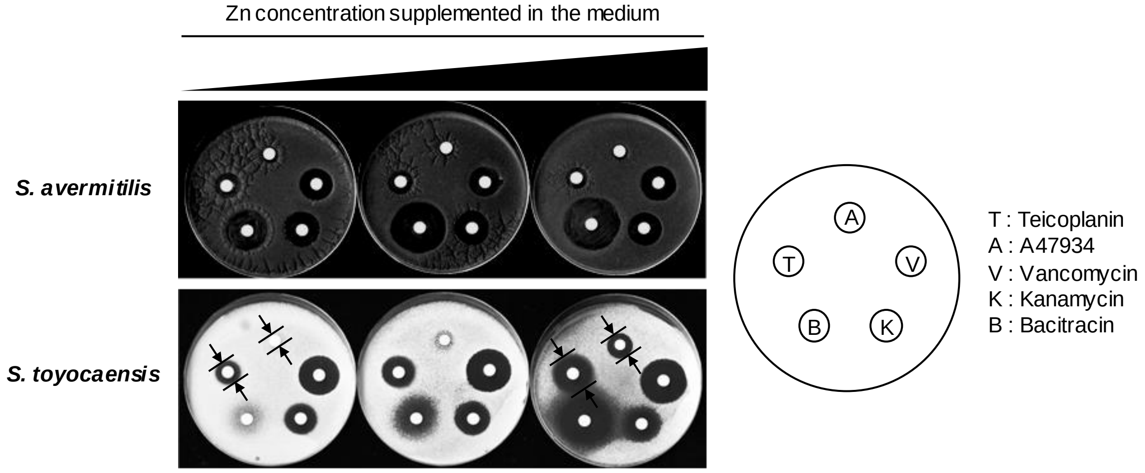

	Ability to induce resistance	Activity influenced by Zn(II)
(T) Tecioplanin	+	+
(A) A47934	+++++	++++
(V) Vancomycin	-	- /+

Supplementary Figure 2: The effect of Zn(II) on the activity of teicoplanin (T), A47934 (A), vancomycin (V), kanamycin (K) and bacitracin (B) against *S. avermitilis* and *S. toyocaensis* strains. *S. avermitilis* does not produce any glycopeptide antibiotic, and no glycopeptide resistance genes are present in the genome, therefore it is totally susceptible to all glycopeptide antibiotics. *S. toyocaensis* is the producer of a sugarless glycopeptide antibiotic, A47934, and the genome carries an A47934 resistance gene cluster adjacent to its synthetic gene cluster. The table summarizes the bioassay results shown above, presenting the influence of Zn(II) on antibiotic activity against an index summarizing the ability of each antibiotic to induce the VanS sensor in *S. toyocaensis*, obtained from previous study[6]. For this assay, *S. avermitilis* was grown on MMCGT agar medium supplemented with up to 200 μM of metal sulphate (0, 100 and 200 μM), while *S. toyocaensis* was grown on up to 100 μM of zinc sulphate (0, 50 and 100 μM).

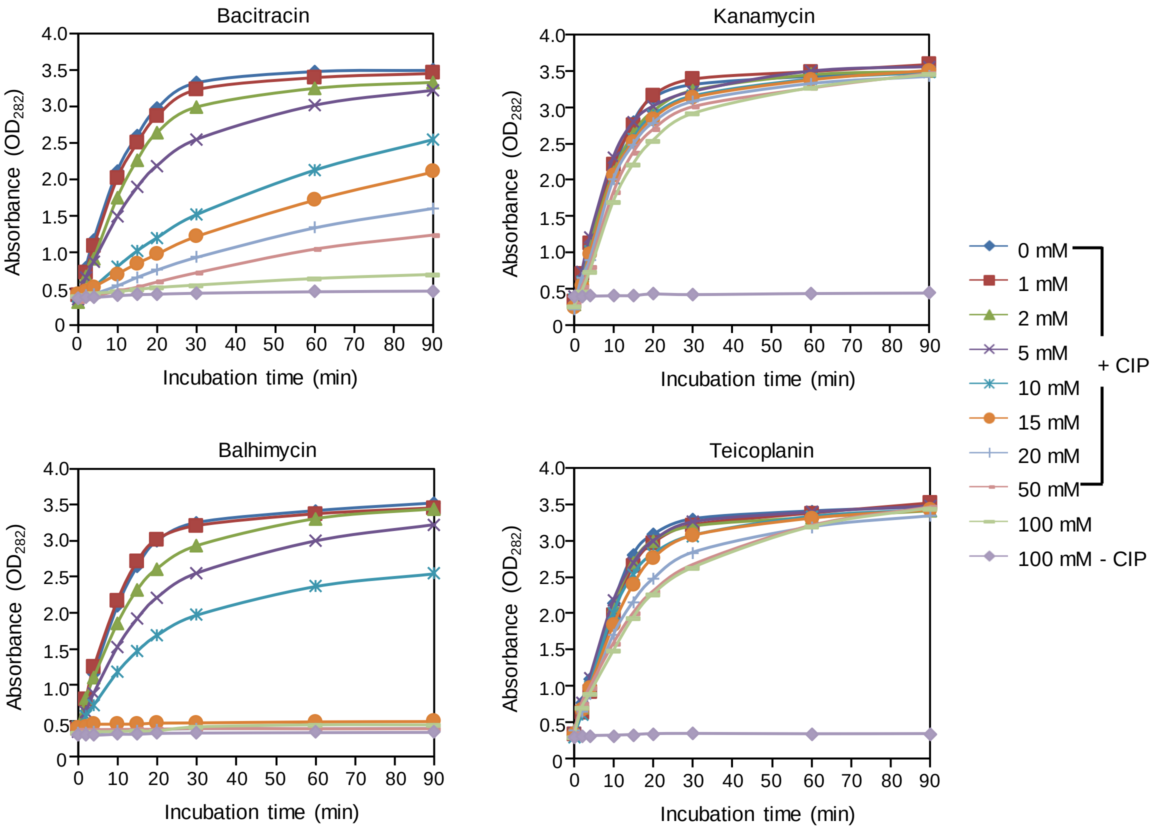

Supplementary Figure 3: The effect of teicoplanin and balhimycin on the activity of CIAP. A CIAP enzyme activity assay indicates the chelation of Zn(II) by balhimycin but not by teicoplanin. 50 µl aliquots of a concentration series (0, 1, 2, 5, 10, 15, 20, 50 and 100 mM) of antibiotic were added to 50 µl aliquots of CIAP (0.1 U in the supplied buffer) then mixed with 100 µl aliquots of pNPP substrate (0.5 mg ml^{-1} in 50 mM Tris-Cl, pH8). A negative control containing antibiotic and pNPP subtrate but without CIAP was also used for each antibiotic tested. The rate of colour evolution is quantified at OD_{415} for 90 min at room temperature. Bacitracin serves as a positive control for this assay as it is known to strongly interact with Zn(II). Kanamycin serves as a negative control as it is not a Zn(II) chelator.

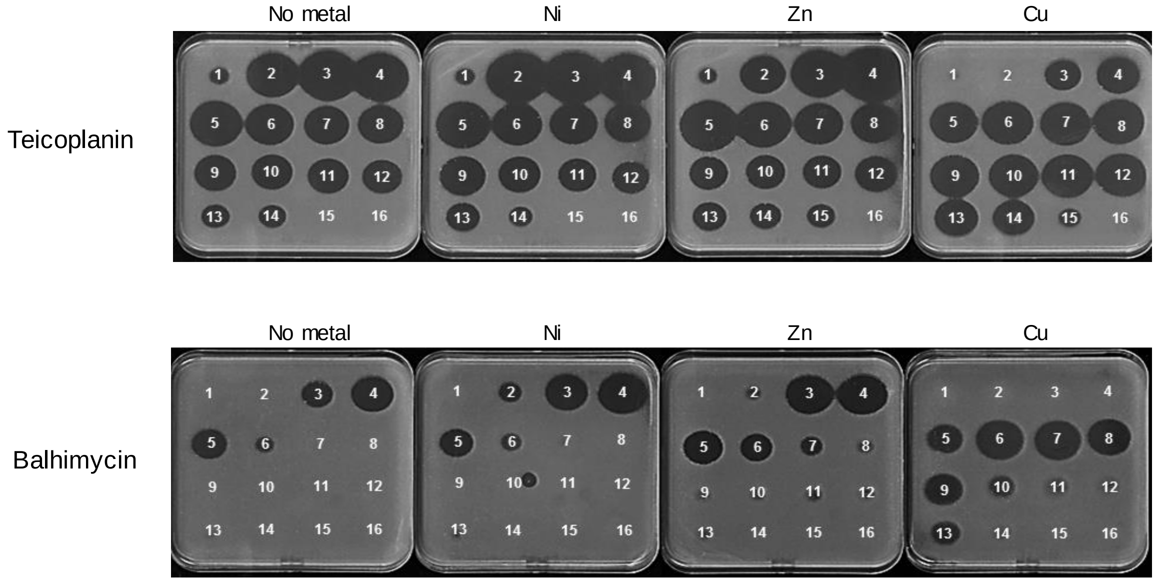

Supplementary Figure 4: Bioassay analysing the elution of teicoplanin and balhimycin from metal affinity chromatography columns. The same column elution fractions analysed spectrophotometrically in Fig. 2b were also analysed by bioassay. A 20 µl aliquot of each diluted fraction was applied to a freshly prepared MMCGT bioassay plates seeded with ~10^7 spores of a vancomycin-sensitive *S. coelicolor* Δ*vanRS* indicator strain. The results were scored after incubation at 30ºC for 2-4 days. A halo of non-growth around the spotted fractions indicates the presence of antibiotic, and halo size is proportional to the amount of antibiotic present in the sample.

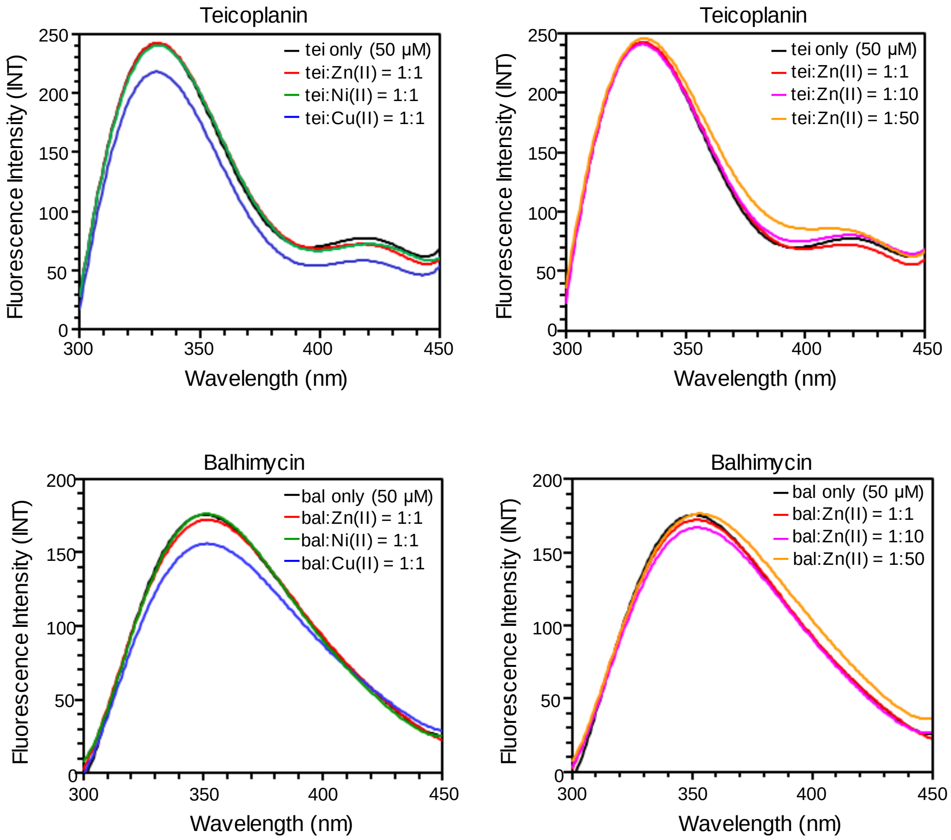

Supplementary Figure 5: Analysis of the interaction of teicoplanin and balhimycin with three selected divalent metal ions using flurometry (λmax (excitation) = 280 nm, Slit: 5 nm, 100 mM Tris-Cl, pH7.3). The fluorescence emission of balhimycin is altered by binding to Cu(II) or Zn(II), but not Ni(II). Teicoplanin similarly interacts with Cu(II) but not Zn(II) or Ni(II). Balhimycin fluoresence is quenched by the addtion of Zn(II) up to a 1:10 molar ratio but enhanced at higher Zn(II) concentrations.

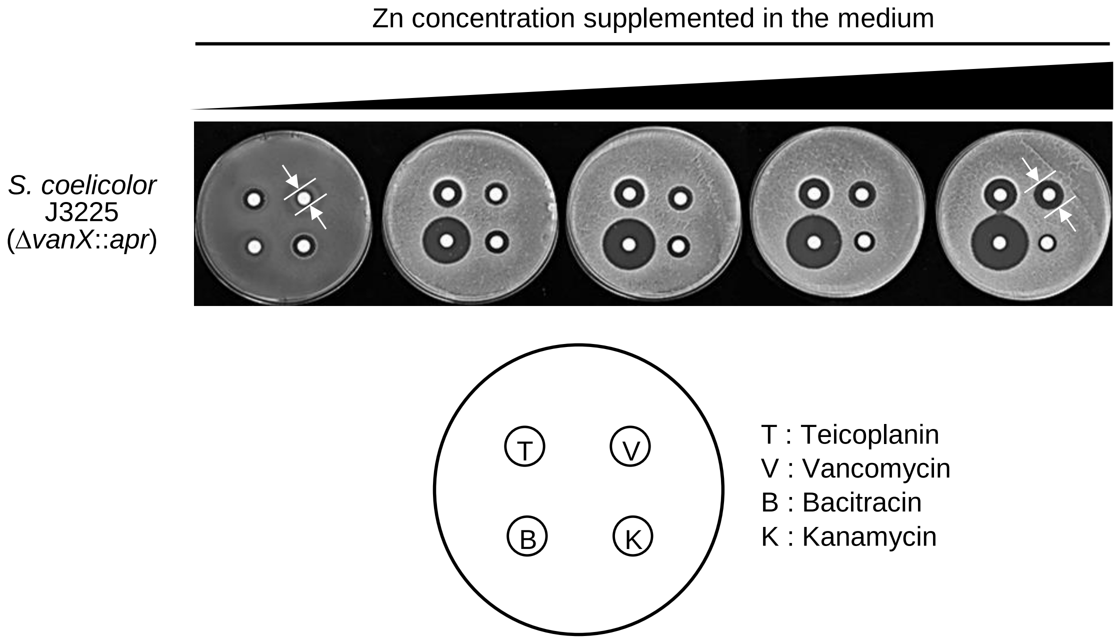

Supplementary Figure 6: The effect of Zn(II) on the activity of teicoplain (T), vancomcyin (V), bacitracin (B) and kanamycin (K) against a Δ*vanX* mutant strain of *S. coelicolor*. Zn(II) also increases the actvity of vancomycin against an *S. coelicolor* Δ*vanX* mutant strain, indicating that an effect of Zn(II) on the activity of VanX does mediate the increase in antibiotic activity. VanX encodes a Zn(II)-dependent D-Ala-D-Lac dipeptidase and is one of the three enzymes, including VanH and VanA, induced by vancomycin and essential for the remodelling of cell wall peptidoglycan biosynthesis that is the basis of resistance to the antibiotic. For this assay, *S. coelicolor* Δ*vanX* was grown on MMCGT agar medium supplemented with up to 200 μM of zinc sulphate (0, 50, 100, 150 and 200 μM).

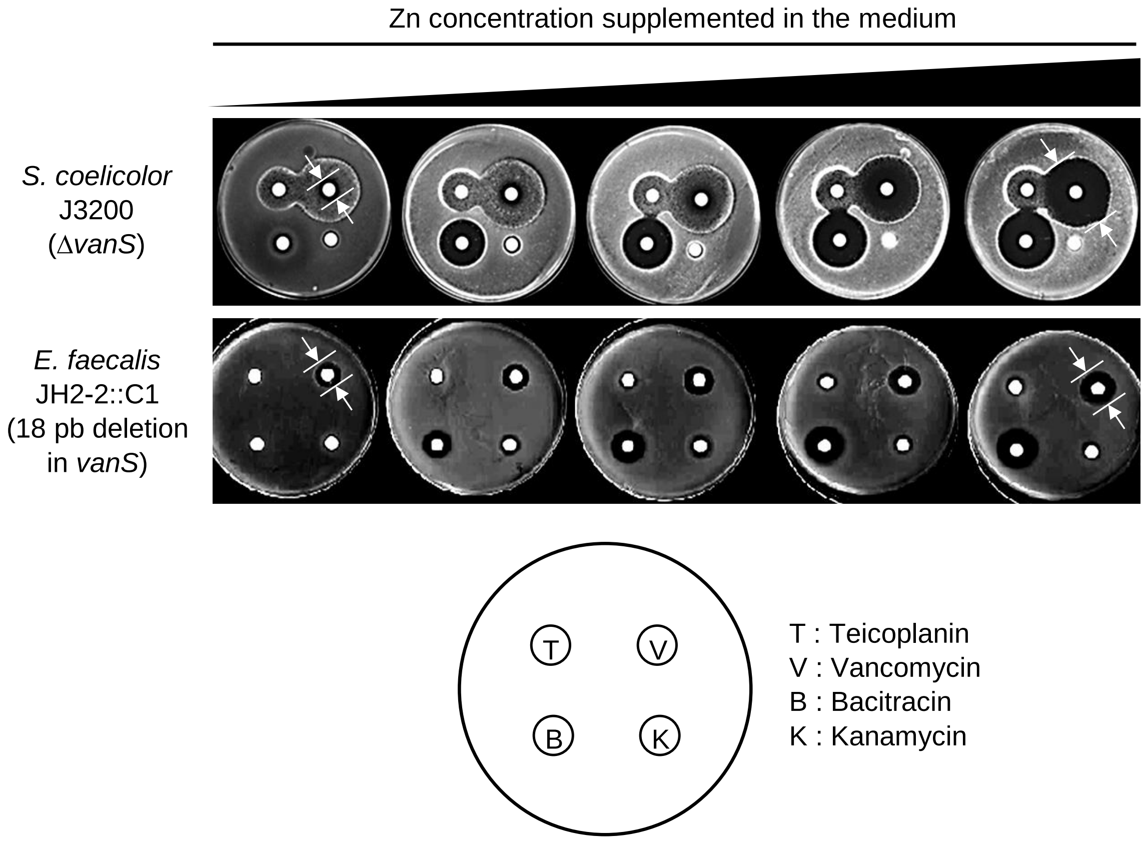

Supplementary Figure 7: The effect of Zn(II) on the activity of teicoplain (T), vancomcyin (V), bacitracin (B) and kanamycin (K) against *vanS* mutant strains of *S. coelicolor* and *E. faecalis*. *vanS* mutant strains (which constitutively express their glycopeptide resistance systems) retain the ability for Zn(II) to positively influence the activity of vancomycin. This implies that the effect of Zn(II) on vancomycin is not mediated by modulation of the sensing by VanS. For this assay, the *S. coelicolor vanS* mutant strain was grown on MMCGT agar medium supplemented with up to 200 µM of zinc sulphate (0, 50, 100, 150 and 200 µM). The *E. faecalis vanS* mutant was grown on up to 400 µM of zinc sulphate (0, 100, 200, 300 and 400 µM).

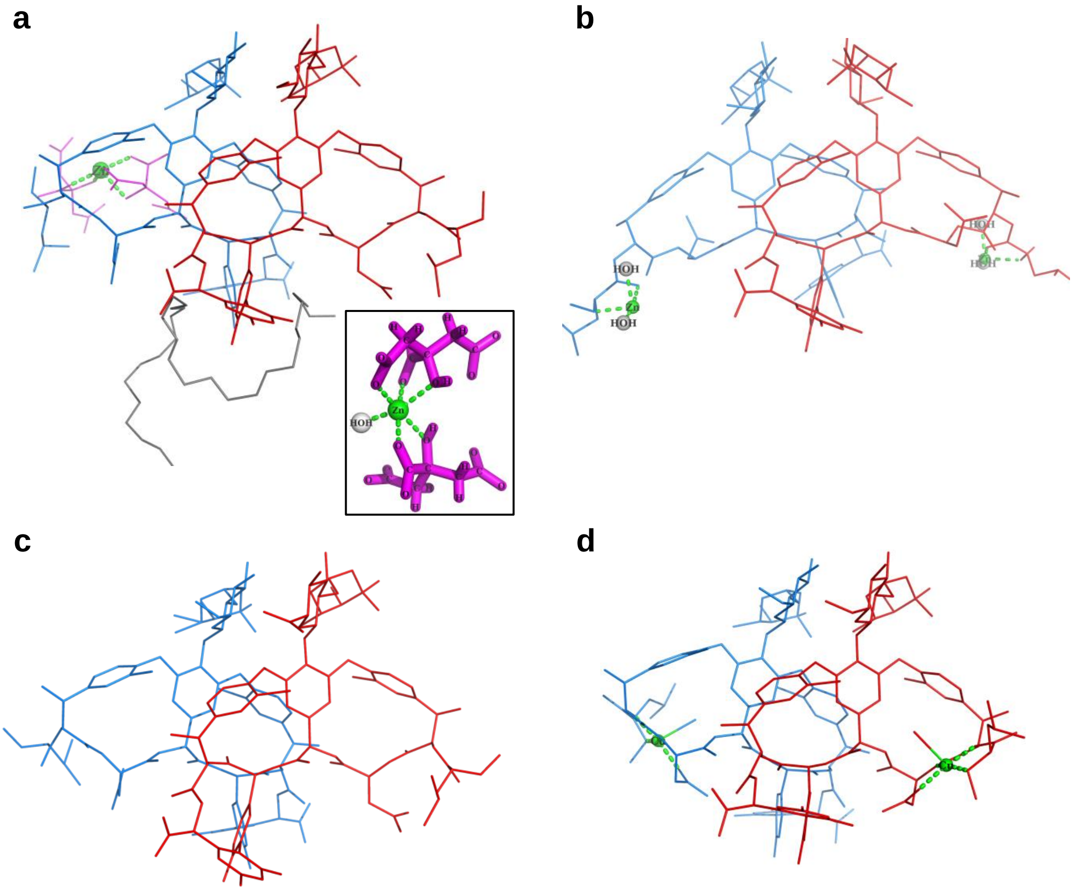

Supplementary Figure 8: Comparison of the vancomycin crystal structures generated in this study (a, b) with previously publisehd structures (c, d). (**a**) Vancomycin dimer in space group C222, in addition to a separate complex of Zn(II) coordinated by two citrate molecules (illustrated separately) and a polyethylene glycol molecule. (**b**) Vancomycin dimer in space group H3 complexed with Zn(II) ions (the asymmetric unit contains one Zn(II) ion only while the other Zn(II) is derived from the next asymmetric unit). (**c**) Vancomycin dimer in space group $P4_32_12$[7]. (**d**) Vancomycin dimer in space group C2 complexed with Cu(II)[8].

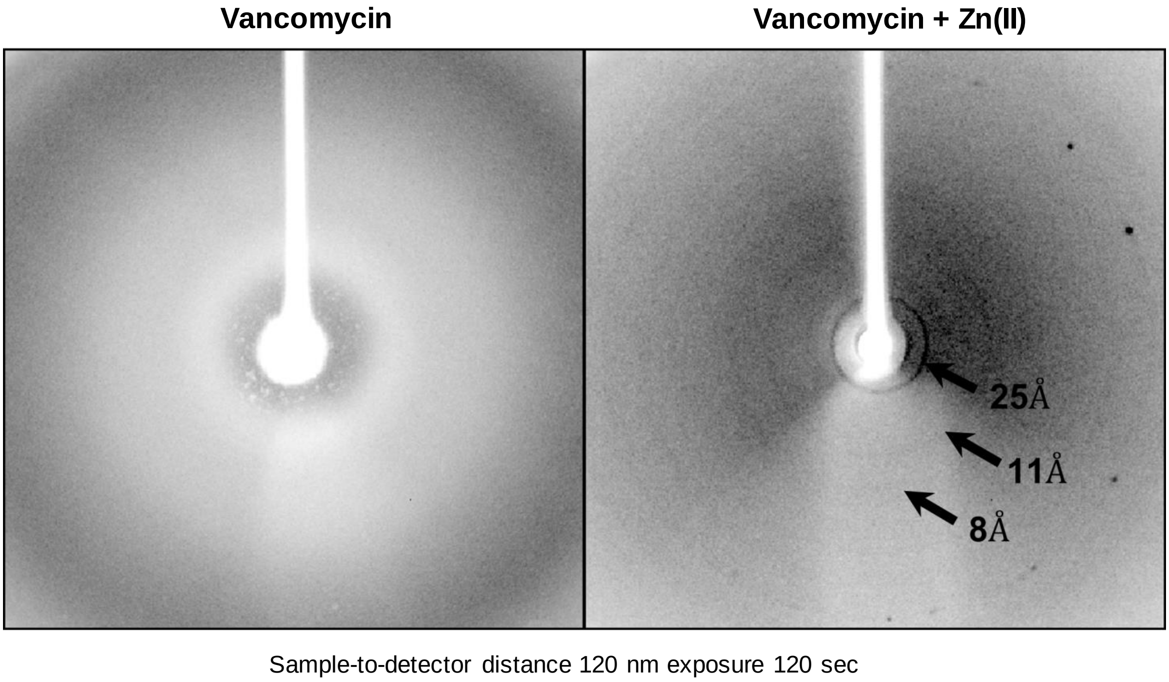

Supplementary Figure 9: X-ray fibre diffraction analysis of fibres generated from vancomycin and a mixture of vancomycin with Zn(II). The sample with Zn(II) shows three main diffraction rings at 25 Å, 11 Å and 8 Å indicative of polymerization that are absent in the vancomycin-only sample.

Supplementary Table 1: Antibiotic concentrations used for the bioassays in this study.

Strain	Concentration of antibiotic used for disc diffusion assay (µg ml^{-1})							
	V	T	B	K	A	Bl	C	R
E. faecalis JH2-2	2	2	10	5	-	-	-	-
E. faecalis JH2-2::I	10	2	10	5	-	-	-	-
E. faecalis JH2-2::C1	10	2	10	5	-	-	-	-
S. coelicolor M600	100	5	5	5	20	100	10	10
S. coelicolor J3200	100	5	5	5	-	-	-	-
S. coelicolor J3201	5	5	5	5	5	5	5	5
S. coelicolor J3225	5	5	5	5	-	-	-	-
S. coelicolor H7300	1	1	10	10	-	-	-	-
S. coelicolor H7400	10	1	10	10	-	-	-	-
S. toyocaensis NRRL15009	5	5	5	5	20	-	-	-
S. avermitilis ATCC31267	5	5	5	5	5	-	-	-

V: vancomycin, T: teicoplanin, B: bacitracin, K: kanamycin, A: A47934, Bl: balhimycin, C: chloroeremomycin, R: ristocetin

Supplementary Table 2: Crystallographic data collection and refinement statistics for the vancomycin crystal, containing a separate complex of Zn(II) coordinated by two citrate molecules, and the vancomycin-Zn(II) crystal.

	Vancomycin crystal	*Vancomycin-Zn(II) crystal*
**Data collection**		
Radiation Source	Diamond (UK), I04-1	Diamond (UK), I04-1
Wavelength (Å)	0.92819	0.92819
Space group	C222	H3
Cell dimensions:		
a, *b*, *c* (Å)	49.04 57.48 21.64	35.90 35.90 55.55
α, β, γ (°)	90 90 90	90 90 120
Resolution (Å)	37.31 - 0.95 (1.00 - 0.95)[1]	27.13 - 1.00 (1.05 - 1.00)
R_{meas} [2] (%)	6.6 (11.9)	8.6 (11.5)
R_{merge}[3] (%)	5.2 (8.5)	6.0 (5.3)
$\langle I / \sigma(I) \rangle$	30.1 (11.7)	27.0 (13.7)
Completeness (%)	98.2 (90.5)	96.3 (81.9)
Redundancy	9.9 (5.3)	8.1 (4.3)
Number of unique reflections	19,399	14,362
**Refinement**		
R_{cryst}[4] / R_{free}[5] (%)	10.5/11.8	9.7/10.5

[1] The statistics shown in parentheses are for the highest-resolution shell.
[2] $R_{meas} = (\boldsymbol{\Sigma}_{hkl} [N/(N-1)]^{1/2} \boldsymbol{\Sigma}_i | I_i(hkl) - I_{mean}(hkl)|) / \boldsymbol{\Sigma}_{hkl} \boldsymbol{\Sigma}_i I_i(hkl)$, where N is redundancy.
[3] $R_{merge} = \boldsymbol{\Sigma}_i | I_i(hkl) - I_{mean}(hkl)| / \boldsymbol{\Sigma}_{hkl} \boldsymbol{\Sigma}_i I_i(hkl)$.
[4] $R_{cryst} = \boldsymbol{\Sigma}_{hkl} ||F_{obs}(hkl)| - |F_{calc}(hkl)||/\boldsymbol{\Sigma}_{hkl} |F_{obs}(hkl)|$
[5] R_{free} is the same as R_{cryst} for a a random subset not included in the refinement of about 10% of total reflection.

Supplementary Table 3: The occurrence of a potential methylleucine Zn(II) binding site in glycopeptide and lipoglycopeptide antibiotic structures, and its relationship to experimentally determined Zn(II) effects.

Antibiotic	Methylleucine group for interaction with Zn(II)	Zn(II) enhances activity in bioassay	Zn(II) binds in vitro
Vancomycin	Yes	Yes	Yes
Teicoplanin	No	No	Weak/No
Ristocetin	No	No	-
A47934	Yes	Yes	-
Chloroeremomycin	Yes	No	-
Balhimycin	Yes	Yes	Yes
Oritavancin	Yes	-	-
Telavancin	Yes	-	-
Dalbavancin	No	-	-

Supplementary Table 4: Plasmids, strains and oligos used in this study.

Plasmid/Strain/Oligo	Plasmid/Strain/Oligo (5'-3')	Reference
<u>Plasmids</u>		
pGN8	*ddlA* gene cloned into pIJ10257 under *ermEp* control (Hyg^R)	9
pGN17	*vanA* gene cloned into pIJ10257 under *ermEp* control (Hyg^R)	9
<u>Strains</u>		
E. coli ET12567 (pUZ8002)	ET12567 containing helper plasmid pUZ8002	10
E. coli BW25113 (pIJ790)	BW25113 containing helper plasmid pIJ790	11
E. faecalis JH2-2	clinical isolate of vancomoycin-sensitive strain	12
E. faecalis JH2-2::I	JH2-2::Tn1549, VanB-type vancomycin-resistant	12
E. faecalis JH2-2::C1	a derivative of JH2-2::I, constitutive vancomycin-resistant strain by 18 bp deletion in *vanS*	12
S. coelicolor M600	SCP1- SCP2-, inducible vancomycin-resistant strain	13
S. coelicolor J3200	Δ*vanS*, SCP1- SCP2-, constitutive vancomycin-resistant strain	14
S. coelicolor J3201	Δ*vanRS*, SCP1- SCP2-, vancomycin-sensitive strain	14
S. coelicolor J3225	Δ*vanX*::*apr*, SCP1- SCP2-	15
S. coelicolor H7100	Δ*vanRS* + *ermEp-ddlA*, SCP1- SCP2-	This study
S. coelicolor H7200	Δ*vanRS* + *ermEp-vanA*, SCP1- SCP2-	This study
S. coelicolor H7300	Δ*vanRS* Δ*ddlA* *ermEp-ddlA*, SCP1- SCP2-	This study
S. coelicolor H7400	Δ*vanRS* Δ*ddlA* *ermEp-vanlA*, SCP1- SCP2-	This study
S. toyocaensis NRRL15009	A47934 producer, A47934-resistant strain	16
S. avermitilis ATCC31267	avermectin producer, glycopeptide-sensitive strain	17
<u>Oligos</u>		
ddlA KO F	tctcgaggcaccgcgggcgggtactctcaacgcgatatgattccggggatccgtcgacc	9
ddlA KO R	gggagtcgccttctctgtggtcacgacacgaaagcgtcatgtaggctggagctgcttc	9
ddlA KO Test F	tgaaggaactgatgtcgcggca	9
ddlA KO Test R	ttcccggaccagacaggaaac	9

Supplementary References

1. Zarkan, A. *et al.* The frontline antibiotic vancomycin induces a zinc starvation response in bacteria by binding to Zn(II). *Sci. Rep.* **6**, 19602 (2016).
2. Bortolato, M., Besson, F. & Roux, B. Role of metal ions on the secondary and quaternary structure of alkaline phosphatase from bovine intestinal mucosa. *Proteins* **37**, 310-318 (1999).
3. Stec. B., Holtz, K. M. & Kantrowitz, E. R. A revised mechanism for the alkaline phosphatase reaction involving three metal ions. *J. Mol. Biol*. **299**, 1303-1311 (2000).
4. Zhang, L., Buchet, R. & Azzar, G. Distinct structure and activity recoveries reveal differences in metal binding between mammalian and *Escherichia coli* alkaline phosphatases. *J. Biochem*. **392**, 407-415 (2005).
5. Morris, K. L. & Serpell, L. C. Chapter 9 X-Ray Fibre Diffraction of Amyloid Fibrils. *Amyloid Proteins - Methods and Protocols*. New York, USA: Springer (2012).
6. Novotna, G., Kwun, M. J. & Hong, H.-J. In vivo characterization of the activation and interaction of the VanR-VanS two-component regulatory system controlling glycopeptide antibiotic resistance in two related *Streptomyces* species. *Antimicrob. Agents Chemother*. **60**, 1627-1637 (2015).
7. Schäfer, M., Schneider, T. R. & Sheldrick, G. M. Crystal structure of vancomycin. *Structure* **4**, 1509-1515 (1996).
8. Marvin, D. A. & Nave, C. X-Ray Fibre Diffraction. *Structural Molecular Biology: Methods and Applications.* New York, USA: Plenum Press (1982).
9. Kwun, M. J. *et al*. In vivo studies suggest that induction of VanS-dependent vancomycin resistance requires binding of the drug to D-Ala-D-Ala termini in the peptidoglycan cell wall. *Antimicrob. Agents Chemother*. **57**, 4470-4480 (2013).
10. Paget, M. S. *et al*. Evidence that the extracytoplasmic function sigma factor σ^E is required for normal cell wall structure in *Streptomyces coelicolor* A3(2). *J. Bacteriol.* **181**, 204-211 (1999).

11. Gust, B. *et al*. PCR-targeted Streptomyces gene replacement identifies a protein domain needed for biosynthesis of the sesquiterpene soil odor geosmin. *Proc. Natl. Acad. Sci. USA* **18**, 1541-1546 (2003).

12. Foucault, M. L., Depardieu, F., Courvalin. P. & Grillot-Courvalin, C*.* Inducible expression eliminates the fitness cost of vancomycin resistance in enterococci. *Proc. Natl. Acad. Sci. USA* **107**, 16964-16969 (2010).

13. Kieser, T. *et al. Practical Streptomyces genetics.* The John Innes Foundation, Norwich (2000).

14. Hutchings, M. I., Hong, H. J. & Buttner, M. J. The vancomycin resistance VanRS signal transduction system of *Streptomyces coelicolor*. *Mol. Microbiol*. **59**, 923-935 (2006).

15. Hong, H. J. *et al*. Characterization of an inducible vancomycin resistant system in *Streptomyces coelicolor* reveals a novel gene (*vanK*) required for drug resistance. *Mol. Microbiol*. **52**, 1107-1121 (2004).

16. Pootoolal, J. *et al*. Assembling the glycopeptide antibiotic scaffold: The biosynthesis of A47934 from *Streptomyces toyocaensis* NRRL15009. *Proc. Natl. Acad. Sci. USA* **99**, 8962-8967 (2002).

17. Waksman, S. A. & henrici, A. T. The Nomenclature and Classification of the Actinomycetes. *J. Bacteriol.* **46**, 337-341 (1943).
